# Identification of Key Genes Involved in Acute Myocardial Infarction by Comparative Transcriptome Analysis

**DOI:** 10.1155/2020/1470867

**Published:** 2020-10-06

**Authors:** Xiaodong Sheng, Tao Fan, Xiaoqi Jin

**Affiliations:** The Department of Cardiovascular Medicine, The Second People's Hospital of Changshu, Jiangsu, China

## Abstract

**Background:**

Acute myocardial infarction (AMI) is regarded as an urgent clinical entity, and identification of differentially expressed genes, lncRNAs, and altered pathways shall provide new insight into the molecular mechanisms behind AMI.

**Materials and Methods:**

Microarray data was collected to identify key genes and lncRNAs involved in AMI pathogenesis. The differential expression analysis and gene set enrichment analysis (GSEA) were employed to identify the upregulated and downregulated genes and pathways in AMI. The protein-protein interaction network and protein-RNA interaction analysis were utilized to reveal key long noncoding RNAs.

**Results:**

In the present study, we utilized gene expression profiles of circulating endothelial cells (CEC) from 49 patients of AMI and 50 controls and identified a total of 552 differentially expressed genes (DEGs). Based on these DEGs, we also observed that inflammatory response-related genes and pathways were highly upregulated in AMI. Mapping the DEGs to the protein-protein interaction (PPI) network and identifying the subnetworks, we found that *OMD* and *WDFY3* were the hub nodes of two subnetworks with the highest connectivity, which were found to be involved in circadian rhythm and organ- or tissue-specific immune response. Furthermore, 23 lncRNAs were differentially expressed between AMI and control groups. Specifically, we identified some functional lncRNAs, including *XIST* and its antisense RNA, *TSIX*, and three lncRNAs (*LINC00528*, *LINC00936*, and *LINC01001*), which were predicted to be interacting with *TLR2* and participate in Toll-like receptor signaling pathway. In addition, we also employed the MMPC algorithm to identify six gene signatures for AMI diagnosis. Particularly, the multivariable SVM model based on the six genes has achieved a satisfying performance (AUC = 0.97).

**Conclusion:**

In conclusion, we have identified key regulatory lncRNAs implicated in AMI, which not only deepens our understanding of the lncRNA-related molecular mechanism of AMI but also provides computationally predicted regulatory lncRNAs for AMI researchers.

## 1. Introduction

Acute myocardial infarction (AMI/MI) is regarded as an urgent clinical entity, whose typical symptoms include pressure and pain in the chest, shortness of breath, sweating, and nausea [1]. In 2017, there were about 10.6 million myocardial infarction cases reported worldwide [2], and MI is still among those top life-threatening conditions and contributed vastly to the hospital admissions and mortality globally [3].

MI can be further divided into ST-segment elevation myocardial infarction (STEMI) and non-STEMI (NSTEMI). Risk factors for MI include high blood pressure, smoking, diabetes, high blood cholesterol, obesity, lack of exercise, and excessive alcohol intake [4], yet critical epicardial coronary disease is absent in approximately 10% of cases of MI occurrence [5]. MI often occurs directly due to the blockage of a coronary artery caused by the rupture or erosion of a vulnerable coronary plaque [5], and its complications cover a wide range including ventricular arrhythmias, cardiogenic shock, stroke, papillary muscle rupture, and pericarditis (Dressler syndrome). While some of these symptoms are present immediately after an MI [6], others might take weeks to develop, and it is challenging for physicians to identify key factors involved in the pathogenesis of MI based on available clinical characteristics [7].

To our knowledge, a variety of genetic factors have been identified to play critical roles in the pathogenesis of ischemic cardiovascular diseases. lncRNAs are an emerging class of noncoding RNAs, which participate in various cellular processes through mechanisms including regulating genomic imprinting and controlling pre-miRNA splicing and mRNA decay [8]. Recent researches have shed some light on how lncRNAs function in the regulation of cardiovascular systems [9, 10]. Moreover, lncRNAs are regarded as more effective tools in distinguishing nonischemic cases from ischemic failing myocardium, compared with the microRNA or mRNA [10]. Several lncRNAs are identified in MI, such as the cyclin-dependent kinase inhibitor 2B antisense RNA 1 (CDKN2B-AS1), member 1 opposite strand/antisense transcript 1 (KCNQ1OT1), myocardial infarction-associated transcript 1 (MIRT1) and 2 (MIRT2), and the lateral mesoderm-specific lncRNA *Fendrr*, which are associated with the activation of the expression of certain genes and capable of reflecting other clinical traits [11–13]. In the present study, we utilized gene expression profiles of circulating endothelial cells (CEC) from 49 patients of acute myocardial infarction (AMI) and 50 controls to identify differentially expressed genes (DEGs), lncRNAs, and pathways, in order to provide promising targets and reveal possible mechanisms behind AMI pathogenesis.

## 2. Material and Methods

### 2.1. Microarray Data and Data Preprocessing

The microarray dataset with accession number GSE66360 [14] was downloaded from the Gene Expression Omnibus (GEO) database (http://www.ncbi.nlm.nih.gov/geo/), which included a total of 99 samples. As reported by a previous study [14], circulating endothelial cells were isolated from patients experiencing acute myocardial infarction (*n* = 49) and from healthy cohorts (*n* = 50). The AMI patients, healthy control patients without a history of chronic disease, and diseased control patients with known but stable cardiovascular disease were aged 18-80, 18-35, and 18-80 years. Refseq IDs labelled as “NR_” were identified as lncRNAs in the Refseq database. To conveniently calculate gene expressions, we used the expression values of probes with the maximal variance to represent the expression of genes matching multiple probes.

### 2.2. Differential Expression Analysis

Following this previous study [15], we used *t*-test and fold change methods to identify differentially expressed genes. To reduce the false-positive rates by multiple testing, BH-adjusted *P* value < 0.05 for *t*-test and fold change between AMI vs. controls > 2 or <1/2 were chosen as the thresholds for differential expression.

### 2.3. Gene Set Overrepresentation Enrichment Analysis

The R package clusterProfiler [16] was used to perform overrepresentation enrichment analysis with *enrichKEGG* function. Terms in the Kyoto Encyclopedia of Genes and Genomes (KEGG) pathways [17] were considered as significantly enriched if the adjusted *P* value < 0.05.

### 2.4. Identification of Subnetwork from Protein-Protein Interaction (PPI)

The protein-protein interactions (PPIs) were extracted from the STRING database [18–20]. The differentially expressed genes (DEGs) were then mapped to the PPI network. The Cytoscape MCODE plugin [21] was applied to search for clustered subnetworks of highly connected nodes from the DEG-based PPI network. The PPI subnetworks were visualized using the Cytoscape software (http://www.cytoscape.org).

### 2.5. lncRNA-Protein Interaction Analysis

The lncRNA-protein interactions were predicted by LncADeep [22], an *ab initio* lncRNA identification and functional annotation tool based on deep learning, as well as the high correlation between the lncRNA and the protein. We used the sequences of differentially expressed lncRNAs and proteins, as well as the correlation between their expression levels, to predict their interactions.

### 2.6. Feature Selection and Support Vector Machine (SVM) Model Construction

To select gene signatures for AMI diagnosis, we employed the MMPC algorithm, which is a constraint-based feature selection algorithm [23]. The 99 samples were first divided into two sets (training (*n* = 50) and validation (*n* = 49)). The features were selected from the model trained using the training set. Based on the selected features, a SVM model was constructed. The SVM model was implemented in R with package e1071. The receiver operating curve (ROC) was generated by the R package ROCR [24].

### 2.7. Statistical Analysis

Statistical comparisons between groups of normalized data were performed using the *t*-test or Wilcoxon rank-sum test according to the test conditions. *P* value < 0.05 was considered to indicate a statistically significant difference with a 95% confidence level. All the statistical analyses were implemented in R (https://www.r-project.org/).

## 3. Results

### 3.1. Identification of Differentially Expressed Genes in AMI

**Figure 1:**
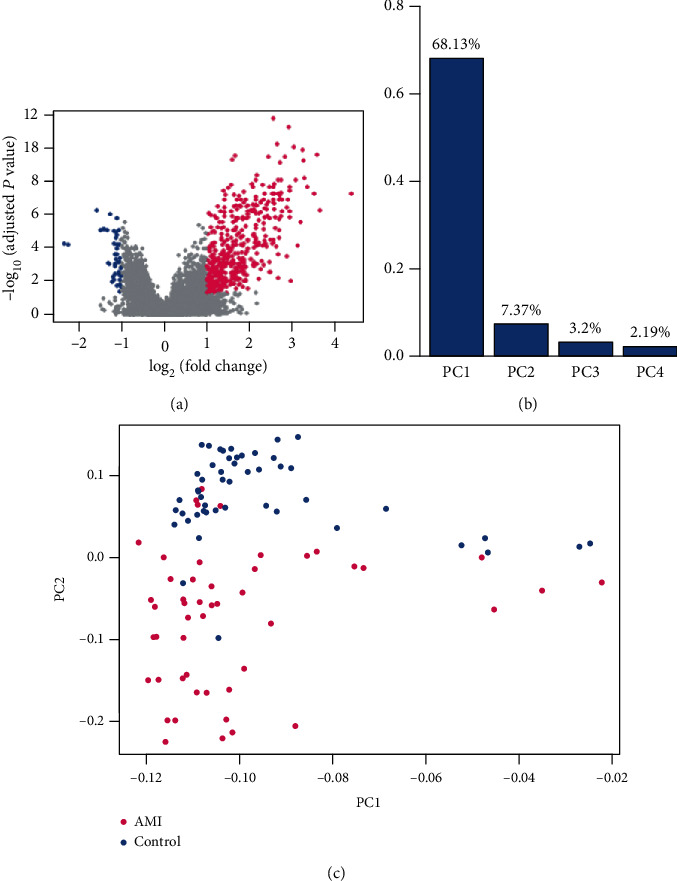
Overview of the differentially expressed genes (DEGs). (a) The upregulated and downregulated genes are colored by red and blue, respectively. (b) The top four principal components (PCs) of the DEGs. (c) The visualization of the samples by first and second PCs. Each point represents one sample, and the AMI cases and controls are represented by red and blue colors, respectively.

With the gene expression profiles of circulating endothelial cells (CEC) from 49 patients of acute myocardial infarction (AMI) and 50 controls, we identified a total of 552 differentially expressed genes (DEGs) (*t*-test, *P* value < 0.05 adjusted by Benjamini and Hochberg (BH), and fold change > 2 or <1/2), including 503 upregulated genes and 49 downregulated genes (Figure 1(a)). Principal component analysis (PCA) revealed that the first four principal components (PCs) accounted for more than 80% of the variance. Particularly, the first PC explained about 68.13% of variance (Figure 1(b)). Moreover, we found that the first two PCs could clearly distinguish the AMI cases from the controls (Figure 1(c)). Moreover, the top ten significantly deregulated genes in AMI included *NR4A2*, *IRAK3*, *NFIL3*, *THBD*, *MAFB*, *IL1R2*, *JUN*, *ACSL1*, *CLEC4E*, and *BCL3* (Table 1). Notably, all these genes were upregulated in AMI. Among the ten genes, *NR4A2*, *IRAK3*, *NFIL3*, *IL1R2*, *CLEC4E*, and *BCL3* were involved in inflammatory response-related biological functions, and *JUN* and *MAFB* were two transcription factors. These results indicated that inflammatory response was an important characteristic of AMI.

### 3.2. Functional Enrichment Analysis of the DEGs

**Figure 2:**
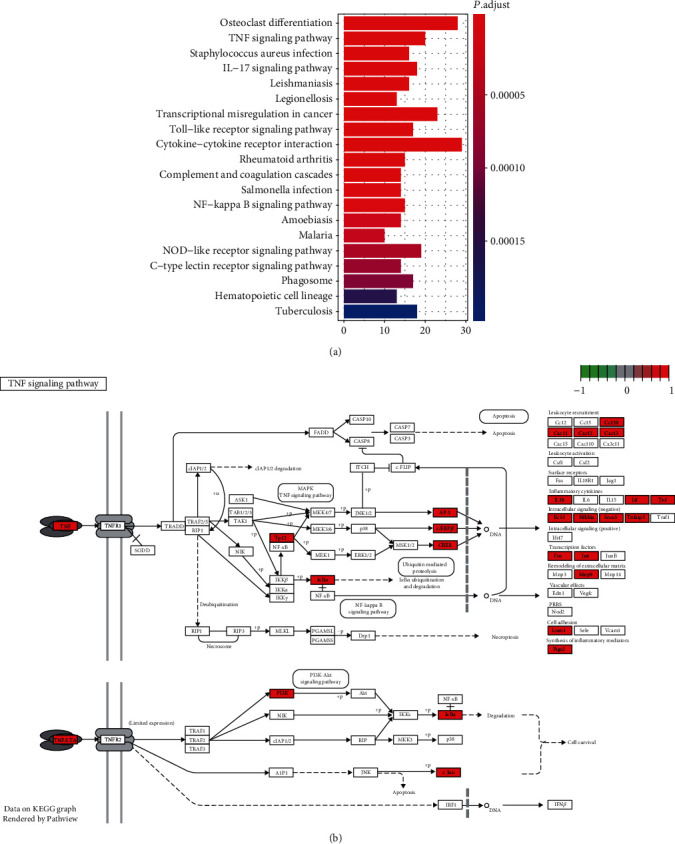
The KEGG pathways enriched by DEGs. (a) The overview of the KEGG pathways enriched by the DEGs. (b) The DEGs involved in the TNF signaling pathway. The upregulated genes were colored by red.

On these differentially expressed genes, the overrepresentation enrichment analysis (ORA) was performed and revealed that inflammatory response-related pathways, including the TNF signaling pathway, IL-17 signaling pathway, Toll-like receptor signaling pathway, cytokine-cytokine receptor interaction, NF-kappa B signaling pathway, and NOD-like receptor signaling pathway, were highly enriched by the upregulated genes (BH-adjusted *P* value < 0.05, Figure 2(a)). However, the downregulated genes were not enriched in any KEGG pathways with the threshold of 0.05 for the BH-adjusted *P* value. Specifically, we further investigated the components involved in the TNF signaling pathway and found that the key transcription factors, such as AP-1 (*JUN* and *FOS*), *CEBPB*, and *CREB5*, as well as their target genes, such as *IL1B*, *LIF*, *TNF*, *BCL3*, *NFKBIA*, *SOCS3*, and *TNFAIP3*, were highly upregulated in AMI patients (Figure 2(b)). These results indicated that the TNF signaling pathway may be a major pathway involved in AMI.

### 3.3. PPI Network Construction

**Figure 3:**
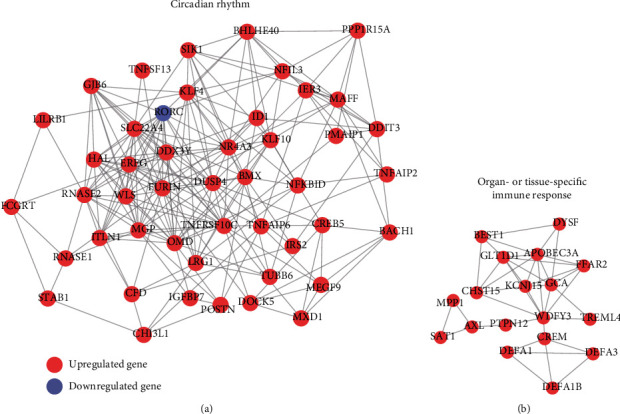
The protein-protein interaction (PPI) subnetworks constructed by the differentially expressed genes. The two PPI subnetworks ((a) and (b)) were identified by the MCODE algorithm. The red and purple nodes represent the upregulated and downregulated genes.

To identify key subnetworks from the protein-protein interaction (PPI) network, we applied the Cytoscape MCODE plugin to search for clustered subnetworks of highly connected nodes from the PPI network. We successfully identified two subnetworks with high connectivity (Figure 3, the Plugin MCODE with the following default parameters: degree cut-off, ≥3; and nodes with edges, ≥3-core, and found that *OMD* (Osteoadherin) and *WDFY3* (WD Repeat and FYVE Domain Containing 3) were the hub genes of the two subnetworks with the highest connectivity. Moreover, the two subnetworks were then found to be involved in circadian rhythm (Figure 3(a)) and organ- or tissue-specific immune response (Figure 3(b)), respectively, suggesting that circadian rhythm and organ- or tissue-specific immune response may be associated with AMI.

### 3.4. Identification of AMI-Associated Long Noncoding RNAs

**Figure 4:**
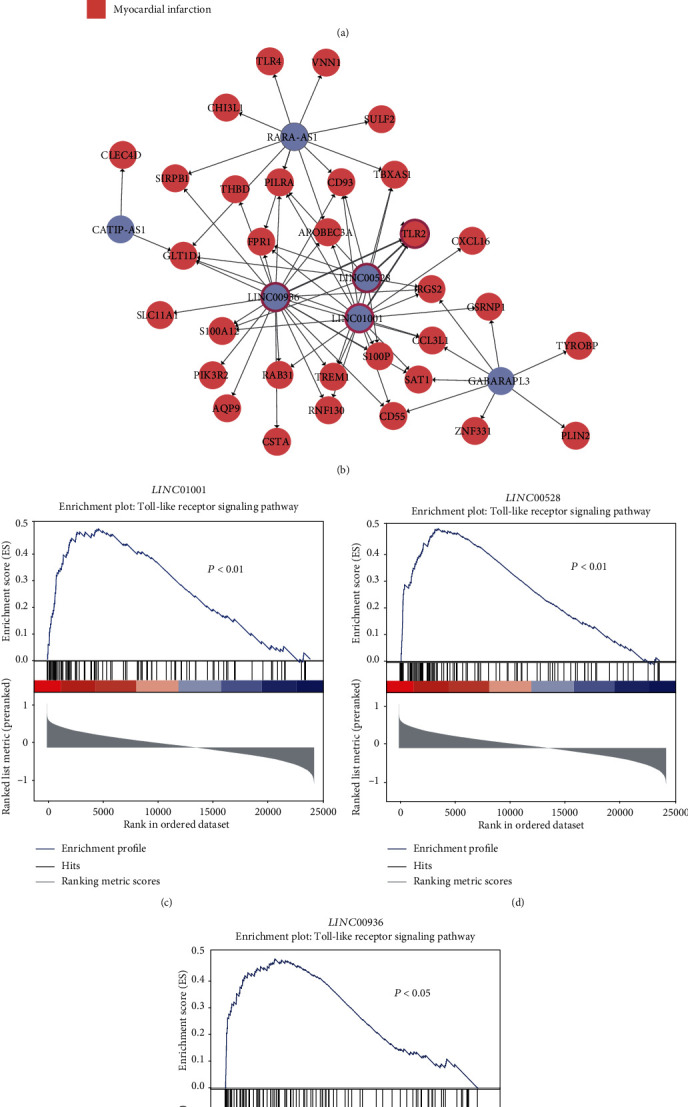
Computational prediction of functional lncRNAs in AMI. (a) The expression profiles of differentially expressed lncRNAs. (b) The lncRNA and protein interaction network. The predicted pathway that the three lncRNAs may participate in is illustrated in (c–e).

In addition to some protein-coding genes (PCGs), some long noncoding RNAs (lncRNAs) could also be quantified using the microarray platform. Based on the gene annotation, we identified 2,242 lncRNAs, 23 of which were differentially expressed between AMI and control groups (Figure 4(a)). Specifically, *XIST* and its antisense RNA *TSIX*, which have been reported to be associated with several diseases [25–27], were significantly downregulated in AMI samples. In accordance with the upregulated genes, the majority of the differentially expressed lncRNAs in AMI samples were the upregulated lncRNAs.

To identify functional lncRNAs that could potentially interact with proteins, we applied a deep learning algorithm, LncADeep [22], to predict the lncRNA-protein interactions. Totally, 71 lncRNA-protein interactions, which consisted of 6 lncRNAs and 32 proteins, were identified and selected based on LncADeep and Pearson correlation coefficient (*r* > 0.6, Figure 4(b)). Notably, *LINC00528*, *LINC00936*, and *LINC01001* were predicted to have interactions with *TLR2* (Toll-like receptor 2). Consistently, the three lncRNAs and *TLR2* were also predicted to participate in the Toll-like receptor signaling pathway (Figures 4(c)–4(e)). These results indicated that these three functional lncRNAs may participate in the pathogenesis of AMI via regulating the Toll-like receptor signaling pathway.

### 3.5. Selection of Gene Signatures for AMI Diagnosis

**Figure 5:**
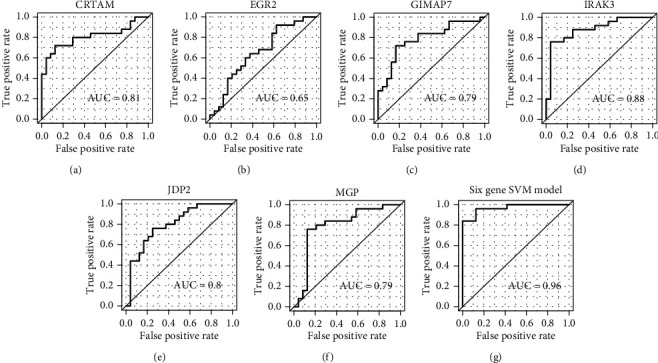
The performance of SVM models built based on the six signature genes. The ROCs of SVM models separately built by six signature genes are illustrated in (a–f). The ROC of the multivariable SVM model based on the six signature genes is displayed in (g).

With the gene expression profiles of circulating endothelial cells (CEC) isolated from whole blood, we then attempted to obtain gene signatures for the classification of AMI and healthy controls. The 99 samples were first randomly divided into training (*n* = 50) and validation (*n* = 49) sets. We identified six gene signatures, including *CRTAM*, *EGR2*, *GIMAP7*, *IRAK3*, *JDP2*, and *MGP*, based on the MMPC algorithm, which identified minimal feature subsets of all the genes from the training set. These six genes were then used to construct six SVM (Support Vector Machine) models based on the training set, separately. The predictive performance of the six models in the validation set revealed that the area under the curve (AUC) of each model was about 0.8, except the SVM built with EGR2 (Figures 5(a)–5(f)). Particularly, the multivariable SVM model based on these six genes achieved the highest performance (AUC = 0.97) as compared with each of these six SVM models. These results suggested that the selected gene signatures could be potential diagnostic biomarkers for AMI.

## 4. Discussion

In the present study, we used gene expression profiles of circulating endothelial cells (CEC) from 49 patients of acute myocardial infarction (AMI) and 50 controls to identify a total of 552 differentially expressed genes (DEGs), including 503 upregulated genes and 49 downregulated genes, and observed that inflammatory response-related genes *NR4A2*, *IRAK3*, *NFIL3*, *IL1R2*, *CLEC4E*, and *BCL3* were highly upregulated in AMI, which was in accordance with the observation that inflammatory response-related pathways were enriched by these upregulated genes, indicating that inflammatory response was one of the important characteristics in AMI. Among the dysregulated KEGG pathways, the TNF signaling pathway was the most significant inflammatory response-related pathway. We found that the key transcription factors, such as AP-1 (*JUN* and *FOS*), *CEBPB*, and *CREB5*, as well as their target genes, such as *IL1B*, *LIF*, *TNF*, *BCL3*, *NFKBIA*, *SOCS3*, and *TNFAIP3*, were highly upregulated in AMI. Notably, some polymorphisms of susceptible genes, key receptors and ligands, and downstream target genes involved in TNF signaling [28–30] have been widely reported by previous studies. When mapping these DEGs to the PPI network, we have identified two PPI subnetworks and found that *OMD* (Osteoadherin) and *WDFY3* (WD Repeat And FYVE Domain Containing 3) were the hub nodes of these two subnetworks with the highest connectivity, which could be involved in circadian rhythm and organ- or tissue-specific immune response. The protein coded by *OMD*, osteomodulin, has been reported to be associated with cardiovascular risk traits [31]. Although *WDFY3* has not been reported to cause AMI, the involvement of *WDFY3* in organ- or tissue-specific immune response further demonstrated its critical role in AMI. Moreover, the circadian rhythm was also associated with AMI [32].

Among the DEGs, 23 lncRNAs were differentially expressed between AMI and control groups. Specifically, *XIST* and its antisense RNA, *TSIX*, which have been reported to be associated with several diseases [25–27], were dominantly downregulated in AMI, suggesting that this pair of lncRNAs may also be responsible for the occurrence of AMI. The predicted interactions between lncRNAs and proteins also highlighted three lncRNAs, namely, *LINC00528*, *LINC00936*, and *LINC01001*, which were predicted to interact with *TLR2* and participate in the Toll-like receptor signaling pathway. As the TLR2 and Toll-like receptor signaling pathway have been reported as a critical regulator and pathway in AMI [33, 34], these lncRNAs may also act as the upstream regulators of this pathway. Recently, *LINC00528* was identified to regulate myocardial infarction by targeting the miR-143-3p/COX-2 axis [35]. Furthermore, we also searched for gene signatures that could discern AMI samples from healthy controls and employed the MMPC algorithm to identify six gene signatures, including *CRTAM*, *EGR2*, *GIMAP7*, *IRAK3*, *JDP2*, and *MGP* for AMI diagnosis. Particularly, the multivariable SVM model based on the six genes achieved high performance (AUC = 0.97), suggesting that these selected gene signatures could be potential diagnostic biomarkers for AMI. Particularly, *EGR2*, a proapoptotic gene, was upregulated in AMI, and its high expression might induce apoptosis in cardiomyocytes [36].

In addition, some limitations also existed in the present study. First, molecular experiments would be needed to validate the biological function of these regulatory lncRNAs. Second, more samples are needed to further validate the performance of the gene signatures for AMI diagnosis. We hope to conduct further research with molecular experiments and more samples in the near future. In conclusion, we have identified key regulatory lncRNAs implicated in AMI and identified six gene signatures in circulating endothelial cells to predict the presence of AMI, which might be useful for the early diagnosis of AMI in clinical application.

## Figures and Tables

**Table 1:** The top ten significantly deregulated genes in AMI.

Gene symbol	*t*-statistic	*P* value	FDR	log_2_FC
*NR4A2*	10.17	7.74*E* − 17	1.89*E* − 12	2.57
*IRAK3*	10.16	5.14*E* − 16	6.29*E* − 12	2.92
*NFIL3*	9.23	7.76*E* − 15	6.32*E* − 11	2.64
*THBD*	9.60	1.60*E* − 14	9.75*E* − 11	3.04
*MAFB*	9.04	2.89*E* − 14	1.41*E* − 10	3.25
*IL1R2*	9.44	6.75*E* − 14	2.75*E* − 10	3.58
*JUN*	8.70	8.56*E* − 14	2.99*E* − 10	1.67
*ACSL1*	8.86	1.30*E* − 13	3.67*E* − 10	2.45
*CLEC4E*	8.88	1.35*E* − 13	3.67*E* − 10	2.84
*BCL3*	8.50	2.38*E* − 13	5.28*E* − 10	1.61

FDR: false discovery rate; log_2_FC: log_2_ fold change.

## Data Availability

The microarray dataset is with accession number GSE66360.

## References

[1] Boateng S., Sanborn T. (2013). Acute myocardial infarction. *Disease-a-Month*.

[2] GBD 2017 Disease and Injury Incidence and Prevalence Collaborators (2018). Global, regional, and national incidence, prevalence, and years lived with disability for 354 diseases and injuries for 195 countries and territories, 1990-2017: a systematic analysis for the Global Burden of Disease Study 2017. *The Lancet*.

[3] Asaria P., Elliott P., Douglass M. (2017). Acute myocardial infarction hospital admissions and deaths in England: a national follow-back and follow-forward record-linkage study. *The Lancet Public Health*.

[4] Mehta P. K., Wei J., Wenger N. K. (2015). Ischemic heart disease in women: a focus on risk factors. *Trends in Cardiovascular Medicine*.

[5] Anderson J. L., Morrow D. A. (2017). Acute myocardial infarction. *The New England Journal of Medicine*.

[6] Reed G. W., Rossi J. E., Cannon C. P. (2017). Acute myocardial infarction. *The Lancet*.

[7] Fox K. A. A., Dabbous O. H., Goldberg R. J. (2006). Prediction of risk of death and myocardial infarction in the six months after presentation with acute coronary syndrome: prospective multinational observational study (GRACE). *BMJ*.

[8] Niland C. N., Merry C. R., Khalil A. M. (2012). Emerging roles for long non-coding RNAs in cancer and neurological disorders. *Frontiers in Genetics*.

[9] Thum T., Condorelli G. (2015). Long noncoding RNAs and microRNAs in cardiovascular pathophysiology. *Circulation Research*.

[10] Yang K. C., Yamada K. A., Patel A. Y. (2014). Deep RNA sequencing reveals dynamic regulation of myocardial noncoding RNAs in failing human heart and remodeling with mechanical circulatory support. *Circulation*.

[11] Vausort M., Wagner D. R., Devaux Y. (2014). Long noncoding RNAs in patients with acute myocardial infarction. *Circulation Research*.

[12] Zangrando J., Zhang L., Vausort M. (2014). Identification of candidate long non-coding RNAs in response to myocardial infarction. *BMC Genomics*.

[13] Grote P., Wittler L., Hendrix D. (2013). The tissue-specific lncRNA Fendrr is an essential regulator of heart and body wall development in the mouse. *Developmental Cell*.

[14] Muse E. D., Kramer E. R., Wang H. (2017). A whole blood molecular signature for acute myocardial infarction. *Scientific Reports*.

[15] MAQC Consortium (2006). The MicroArray Quality Control (MAQC) project shows inter- and intraplatform reproducibility of gene expression measurements. *Nature Biotechnology*.

[16] Yu G., Wang L. G., Han Y., He Q. Y. (2012). clusterProfiler: an R package for comparing biological themes among gene clusters. *OMICS*.

[17] Kanehisa M., Goto S. (2000). KEGG: kyoto encyclopedia of genes and genomes. *Nucleic Acids Research*.

[18] Szklarczyk D., Morris J. H., Cook H. (2017). The STRING database in 2017: quality-controlled protein-protein association networks, made broadly accessible. *Nucleic Acids Research*.

[19] Shi X., Huang T., Wang J. (2018). Next-generation sequencing identifies novel genes with rare variants in total anomalous pulmonary venous connection. *eBioMedicine*.

[20] Gu C., Shi X., Huang Z. (2020). A comprehensive study of construction and analysis of competitive endogenous RNA networks in lung adenocarcinoma. *Biochimica et Biophysica Acta (BBA) - Proteins and Proteomics*.

[21] Shannon P., Markiel A., Ozier O. (2003). Cytoscape: a software environment for integrated models of biomolecular interaction networks. *Genome Research*.

[22] Yang C., Yang L., Zhou M. (2018). LncADeep: an ab initio lncRNA identification and functional annotation tool based on deep learning. *Bioinformatics*.

[23] Brown L. E., Tsamardinos I., Aliferis C. F. (2004). A novel algorithm for scalable and accurate Bayesian network learning. *Studies in Health Technology and Informatics*.

[24] Sing T., Sander O., Beerenwinkel N., Lengauer T. (2005). ROCR: visualizing classifier performance in R. *Bioinformatics*.

[25] Chow J., Heard E. (2009). X inactivation and the complexities of silencing a sex chromosome. *Current Opinion in Cell Biology*.

[26] Wang Z., Jinnin M., Nakamura K. (2016). Long non-coding RNA TSIX is upregulated in scleroderma dermal fibroblasts and controls collagen mRNA stabilization. *Experimental Dermatology*.

[27] Migeon B. R., Chowdhury A. K., Dunston J. A., McIntosh I. (2001). Identification of TSIX, encoding an RNA antisense to human XIST, reveals differences from its murine counterpart: implications for X inactivation. *American Journal of Human Genetics*.

[28] Nilsson L., Szymanowski A., Swahn E., Jonasson L. (2013). Soluble TNF receptors are associated with infarct size and ventricular dysfunction in ST-elevation myocardial infarction. *PLoS One*.

[29] Schulz R., Heusch G. (2009). Tumor necrosis factor-alpha and its receptors 1 and 2: yin and yang in myocardial infarction?. *Circulation*.

[30] Tian M., Yuan Y. C., Li J. Y., Gionfriddo M. R., Huang R. C. (2015). Tumor necrosis factor-*α* and its role as a mediator in myocardial infarction: a brief review. *Chronic Diseases and Translational Medicine*.

[31] Ngo D., Sinha S., Shen D. (2016). Aptamer-based proteomic profiling reveals novel candidate biomarkers and pathways in cardiovascular disease. *Circulation*.

[32] Holmes D. R., Aguirre F. V., Aplin R. (2010). Circadian rhythms in patients with ST-elevation myocardial infarction. *Circulation: Cardiovascular Quality and Outcomes*.

[33] Ha T., Liu L., Kelley J., Kao R., Williams D., Li C. (2011). Toll-like receptors: new players in myocardial ischemia/reperfusion injury. *Antioxidants & Redox Signaling*.

[34] Hally K. E., la Flamme A. C., Larsen P. D., Harding S. A. (2017). Platelet Toll-like receptor (TLR) expression and TLR-mediated platelet activation in acute myocardial infarction. *Thrombosis Research*.

[35] Liu K., Zhao D., Wang D. (2020). LINC00528 regulates myocardial infarction by targeting the miR-143-3p/COX-2 axis. *Bioengineered*.

[36] Tang Y., Wang Y., Park K. M. (2015). MicroRNA-150 protects the mouse heart from ischaemic injury by regulating cell death. *Cardiovascular Research*.

